# Genome-scale modeling and human disease: an overview

**DOI:** 10.3389/fphys.2014.00527

**Published:** 2015-01-23

**Authors:** Matthew A. Oberhardt, Erwin P. Gianchandani

**Affiliations:** ^1^Department of Molecular Microbiology and Biotechnology, Faculty of Life Sciences, School of Computer Sciences, and Sackler School of Medicine, Tel Aviv UniversityTel Aviv, Israel; ^2^Division of Computer and Network Systems, United States National Science FoundationArlington, VA, USA

**Keywords:** genome-scale models, systems analysis, genome-scale metabolic reconstruction, human diseases, systems biology

**A commentary on**

**Genome-scale modeling and human disease**

by Gianchandani, E. P., and Oberhardt, M. A.

The last several decades have seen extraordinary progress in the biomedical sciences. The explosion of sequencing and high-throughput data is both welcome and daunting for the study of human disease: while human disease is increasingly understood to be multi-factorial and systemic, the sheer complexity of the data being generated makes unaided interpretation nearly impossible. Meanwhile, genome-scale modeling (GSM) has emerged as a major scaffold and toolkit for contextualizing rich data, and one especially suited to the thousands-of-datapoints-per-measurement reality of contemporary disease research.

The archetypal genome-scale model is the genome-scale metabolic reconstruction (GENRE), a predictive network model that contains up to several thousand metabolic reactions, as well as associated genes and enzymes (but not kinetics, due to the scale) (Oberhardt et al., 2009). Recently available GENREs of human metabolism have opened up enormous avenues in disease research (Duarte et al., 2007; Ma et al., 2007; Thiele et al., 2013), especially when integrated with high-throughput data [for an extensive review, see in this topic: (Blazier and Papin, 2012)]. These models rely on extensive manual curation, and annotating understudied or ambiguous parts of metabolism is critical for improving their predictive power. In an effort to address one of the most difficult-to-annotate areas of metabolism, researchers involved in the human metabolic reconstruction efforts have provided for this topic a large analysis of membrane transporters in human metabolism, including a discussion of how transport impacts multiple human diseases (Sahoo et al., 2014). GENREs are contributing to many areas of disease research, as detailed below, and their scope and influence will increase as a result of such contributions.

Systemic metabolic disorders such as obesity and diabetes exact a huge toll in the US and worldwide, and GSMs are increasingly being used for their study. Large-scale models of mitochondria, for example, have helped examine obesity-associated aberrations in mitochondrial fatty acid degradation (Van Eunen et al., 2013) and many other aspects of energy metabolism as reviewed in this topic: (Sangar et al., 2012). Similarly, the human GENRE has been used in a number of studies relevant to metabolic diseases [e.g., building a model of human adipocyte—(Mardinoglu et al., 2013); determining biomarkers for inborn errors of metabolism—(Shlomi et al., 2009)], as extensively reviewed here: (Varemo et al., 2013). GENREs are obvious choices for studying metabolically-based diseases, and will likely be relied on more in the future.

Another area of increasing interest in human disease is the impact of the microbial organisms that cohabitate our bodies, collectively known as our “microbiome.” The gut microbiome, for example, has been shown to alter the metabolism of many drugs (Kang et al., 2013), and to be a causative factor in maintaining obese or healthy states (Turnbaugh et al., 2006). GENREs have been used to examine prominent members of the gut microbiota (Heinken et al., 2013), to understand interactions between gut microbes (Shoaie et al., 2013), and to explore interactions between gut microbes and epithelial cells (Sahoo and Thiele, 2013). GSMs are still severely limited in this arena due to challenges in community microbial modeling. However, large-scale microbiome modeling efforts will likely have increasing impact as they mature in the coming years, both by driving new knowledge of complex community phenotypes (e.g., Freilich et al., 2011 and reviewed generally in Greenblum et al., 2013) and by including so-far neglected areas such as the oral microbiome, as reviewed in this topic: (Dimitrov and Hoeng, 2013).

Cancer is a complex and multifaceted disease, and a hallmark for huge data collection efforts. As such, it is a natural target for systems modeling [for a general review of systems biology approaches, see in this topic: (Hernandez Patino et al., 2012)]. Metabolic deregulation in cancer has generated considerable interest within the genome-scale metabolic modeling community, resulting in a number of cancer-related metabolic reconstructions being recently published [see a review in this topic: (Lewis and Abdel-Haleem, 2013)]. Models of specific cancer subtypes are now being built based on the generic human GENRE (Jerby et al., 2010; Agren et al., 2012), and in a few cases, they have revealed insights with therapeutic potentiality (Frezza et al., 2011; Agren et al., 2014).

Due to their lack of kinetic parameters, GENREs alone cannot predict dynamic cell states, nor, surprisingly, can they integrate metabolite concentration data into basic kinetics or allosteric regulation. Since kinetic parameters are difficult to measure and can vary between conditions or cells, ensemble modeling was recently used to estimate kinetic models of human colorectal adenocarcinoma cell lines, and to reveal potential synthetic lethal interactions that could yield new drug targets [see in this topic: (Khazaei et al., 2012)]. Cancer is also a disease marked by the evolutionary process that the cancerous cells undergo. Genomic data and increasingly sophisticated population models are now enabling elucidation of these processes, which are critical for establishing the basis for cures [see a review in this topic: (Stransky and De Souza, 2012)]. These areas have gained a lot of interest, and we expect many more systems-level studies of cancer in the near future.

By contrast, neurological disorders constitute a set of diseases that have not received as much attention in the GSM community, despite the significant impacts illustrated in Figure 1. Early attention focused on genome-wide expression analyses and gene-interaction networks, often using yeast pathways conserved in humans and implicated in neurodegenerative diseases such as Parkinson's, Alzheimer's, and Huntington's (Petranovic and Nielsen, 2008; Noorbakhsh et al., 2009; Wall et al., 2009). More recently, efforts have begun to employ GSM with success. For example, (Lewis et al., 2010) integrated gene expression data, proteomics data, and literature-based manual curation to model brain energy metabolism and recapitulate the metabolic interactions between astrocytes and various neuron types relevant to Alzheimer's disease. In addition, transcriptomic data from Alzheimer's patients were integrated with a genome-scale computational human metabolic model to characterize the altered metabolism in the diseased state, and metabolic modeling methods were employed to predict metabolic biomarkers and drug targets (Stempler et al., 2014). We expect interest in neurological illnesses to continue to rise.

**Figure 1:**
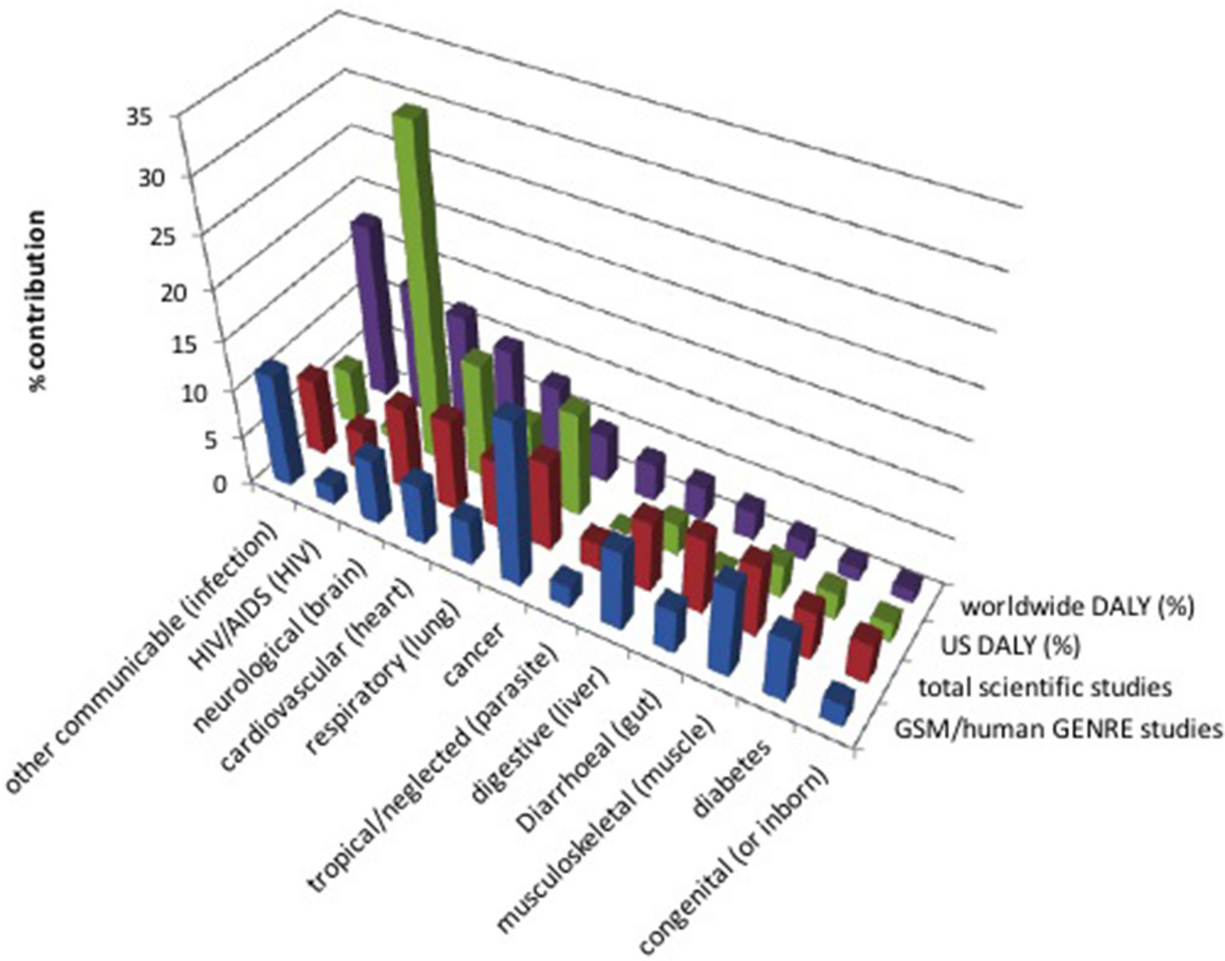
**Publications in different disease areas vs. morbidity rates**. Age standardized disability adjusted life years (DALY), a measure of years of life lost due to disease, is reported as percent worldwide and in the US. Injury (11.3%) and “other non-communicable disease” (7.4%) are not listed due to lack of related search terms. The count of scientific studies found in Google Scholar searches [normalized to 83% (=100% − 11.3% − 7.4%)] of disease-related search terms (listed in parentheses, if different than the disease name) are also shown, along with studies specifically related to genome-scale models or to the human GENRE [the numbers of hits were averaged between hits including the searchterm AND citing (Duarte et al., 2007), and hits including the searchterm AND including the phrase “genome scale model,” before normalization]. DALY data were taken from the World Health Organization (WHO) report for the year 2002 (which is the most updated WHO source of DALYs of which these authors are aware) (Mathers et al., 2005).

While much of this short review focuses on GENRE-based analyses, GENREs are by no means the only genome-scale models of note. Many alternative topology-based methods for pathway analysis are available and have been reviewed here: (Mitrea et al., 2013). We also include in this topic a promising new Boolean-based model for somatic cell reprogramming: (Flottmann et al., 2012). Somatic cell reprogramming is a new and highly promising field—it first emerged in 2006 with the landmark paper (Takahashi and Yamanaka, 2006)—that could lead to novel therapeutic approaches, such as growing organs from skin cells for self-transplant.

GSM-based analysis is now a key asset in studying disease. The works in this topic reflect trends in the biomedical sciences at large, including areas of intense interest (e.g., cancer) as well as those that have been labeled as neglected diseases (e.g., few models have been built for studying parasitic tropical diseases or HIV/AIDS). Although eukaryote reconstructions are more challenging due to genome sizes, knowledge coverage, and the multitude of cellular compartments (Thiele and Palsson, 2010), we expect the successes described in this overview to continue to mount—with a particular focus in coming years on clinical problems with translatable outcomes, in which models will help identify new drug targets or alternate cures. This is already evident from recent DREAM Challenges, which have sought to foster collaboration and build communities around fundamental questions at the intersection of systems biology and translational medicine [see, for example, (Margolin et al., 2013)]. To help guide and contextualize disease study, we have included a chart of the most devastating diseases, along with the amount of focus in GSM studies as well as in science at large toward addressing them (Figure 1). Shifting focus toward neglected areas is a worthy goal to which we hope this mini-review will contribute.

## Conflict of interest statement

There are no commercial or financial conflicts of interest. However, the views expressed in this article are strictly those of EPG and not representative of those of the US National Science Foundation.
